# Oral Administration of Coronavirus Spike Protein Provides Protection to Newborn Pigs When Challenged with PEDV

**DOI:** 10.3390/vaccines9121416

**Published:** 2021-11-30

**Authors:** Magdalena Maj, Gina M. Fake, John H. Walker, Ryan Saltzman, John A. Howard

**Affiliations:** 1 Department of Biological Sciences, California Polytechnic State University, San Luis Obispo, CA 93407, USA; mmaj@calpoly.edu; 2 Applied Biotechnology Institute, Cal Poly Tech Park, San Luis Obispo, CA 93407, USA; gfake@appliedbiotech.org; 3 Department of Statistics, California Polytechnic State University, San Luis Obispo, CA 93407, USA; jwalker@calpoly.edu; 4 Veterinary Resources Inc., Ames, IA 50010, USA; vetres2@msn.com

**Keywords:** PEDV, spike protein, oral delivery, passive immunity, cytokines, GM-CSF, plant-produced vaccines, maize

## Abstract

To investigate whether oral administration of maize-produced S antigen can provide passive immunity to piglets against porcine epidemic diarrhea virus (PEDV), 16 pregnant sows were randomly assigned to one of four treatments: (1) injection of PEDV vaccine (INJ), (2) maize grain without S protein (CON), (3) maize grain containing low dose of S antigen (LOV) and (4) maize grain containing a high dose of S antigen (HOV). Vaccines were administered on days 57, 85 and 110 of gestation. Sows’ serum and colostrum were collected at farrowing and milk on day 6 post-challenge to quantify neutralizing antibodies (NABs) and cytokines. Piglets were challenged with PEDV 3–5 d after farrowing, and severity of disease and mortality assessed on day 11 post-challenge. Disease severity was lower in LOV and INJ compared with HOV and CON, whereas the survival rate increased in piglets from LOV sows compared with HOV and CON (*p* ≤ 0.001). Higher titers of NABs and lower levels of cytokine granulocyte-macrophage colony-stimulating factor in sows’ milk were positively correlated with piglet survivability (*p* ≤ 0.05). These data suggest that feeding S protein in corn to pregnant sows protects nursing piglets against PEDV.

## 1. Introduction

Coronaviruses have become a major problem for both human and animal welfare and are a continuing threat for the future. Porcine epidemic diarrhea virus (PEDV) is a positive strand enveloped RNA virus of family *Coronaviridae* with a genome of 28 kb. The virus infects swine, resulting in major losses to the industry in the U.S. and worldwide [1,2]. Newborn piglets are especially susceptible, with a high mortality rate reaching up to 100% within 7 d after birth [3]. PED virus replicates in the mature intestinal enterocytes leading to villus atrophy and enteritis, causing malabsorptive diarrhea and vomiting [3,4]. The disease was first identified in Europe in the early 1970s, in Asia in 2010 and in the United States in 2013, and it continues to be a major problem in the swine industry worldwide [2,5]. The conditionally approved vaccines in North America from Harrisvaccines (Ames, IA, USA) and Zoetis (Parsippany, NJ, USA) are based on RNA or inactivated virus but are only marginally effective [6,7]. Therefore, there is an urgent need for a more effective vaccine for PEDV. 

The PEDV spike (S) protein is a viral glycoprotein responsible for receptor binding and fusion of host cell receptors, which plays a critical role in the early steps of infection [8]. S protein is the primary immunogen due to its multiple neutralizing epitopes, the major target of neutralizing antibodies, and a likely vaccine candidate [9,10]. Several prototype candidates based on different portions of the spike protein have shown promising immune responses in animal studies [7,11]. These include immunogens based on the S1 moiety [11], the S2 moiety [12], and a smaller portion known as the core neutralizing epitope or COE (amino acids 499–638) that has been identified as containing neutralizing epitopes [13]. However, the prototype vaccines require the purification of the S protein, which has been difficult to produce at high levels in several recombinant systems [11,14,15]. 

Because PEDV initiates its infectious cycle at the intestinal mucosal epithelial surface [16], effective protection would optimally require vaccination which elicits an immune response at both the systemic and mucosal levels [17]. An orally administered vaccine may provide a more robust mucosal response than intramuscular counterparts, and may greatly facilitate widespread vaccination against PEDV by eliminating the need for injections and individual handling of the pigs. Precedent for oral immunization for PEDV includes studies expressing PEDV S or N proteins in probiotics such as *Lactobacillus*. Oral delivery of these products elicits an immune response [18] and protection upon challenge [19]. The S protein for PEDV has been produced in plants including tobacco and rice and has been shown to elicit neutralizing antibodies against the virus [20,21,22,23]. Ideally, protection could be achieved in a system in which the antigen is stable during production, storage and transport, and does not require purification of the antigen away from other toxic compounds.

Maize grain has emerged as a preferred option for oral vaccines as it provides high levels of accumulation of the recombinant proteins [24] and bioencapsulation of the protein to protect it from degradation in the digestive tract [25,26]. The maize system also has many inherent properties, such as stability of recombinant proteins that retain activity for years in the grain, allowing for long-term storage, transport at ambient temperatures, and processing of the grain at will rather than a requirement to process large batches immediately upon harvest [27]. Maize is a major component of feed, providing a safe and non-diluted matrix for delivery and making it amenable to create a practical low-cost oral vaccine for livestock. 

Previous studies with a spike protein from a different coronavirus, porcine transmissible gastroenteritis virus (TGEV), demonstrated that an orally delivered maize-based candidate vaccine elicited an immune response and provided protection upon a challenge in young pigs [28] and stimulated NABs in sows [29]. Other maize-based vaccines have also shown efficacy in animal trials [30] and safety in a human clinical trial [31]. Nonetheless, there is only one report expressing the core neutralizing epitope (COE) domain of the PEDV spike protein in maize, which was subsequently purified and injected in mice to elicit antibodies against PEDV [32]. Our previous work demonstrated that the S1 antigen could accumulate to high levels in maize and allow for a heat-stable, low-cost production supply of the antigen [33]. In addition, 27-day-old pigs fed maize-produced S1 protein developed high levels of sera neutralization antibodies after being challenged with active PED virus [33]. However, because the disease symptoms are acute only in nursing pigs, protection from the virus could not be evaluated in this study. In this report, we administered the S1 vaccine candidate to naïve sows in gestation, and then we challenged their litters with the PED virus 3–5 d after farrowing to investigate whether S protein in corn may provide passive immunity and protection to suckling piglets. 

## 2. Materials and Methods

### 2.1. Production of Maize Expressing S1 Antigen and Preparation of the Corn Material

A transgenic maize line expressing high levels of S1 protein (#PDC18) [33] was grown to obtain grain for this study. Briefly, expression of S1 protein was targeted to the corn embryo, which enabled the S1 antigen to be concentrated by enriching for the germ fraction using a CIM-8-MIS pin mill (Munson; Utica, NY, USA), DP650-14 roller mill (Roskamp; Waterloo, I), and a LS18S55 separator (SWECO; Florence, KY, USA). Germ and grits were dried to a moisture content of less than 12% and ground on a Glen Mill grinder (Clifton, NJ, USA) to obtain corn meal so that >80% of the material could pass through a 20-mesh screen. The expression of the S1 protein was confirmed in the corn meal by Western blotting analysis, following the protocol described in [33]. Transformed corn meal was packed in 1 kg labeled bags and stored at room temperature. Untransformed corn was used to feed the control animals. The low-dose oral vaccine contained approximately 10 mg of S1 antigen/kg corn, and the high-dose oral vaccine contained 50 mg/kg corn. These values were calculated based on Western blotting images of corn meal compared to a purified recombinant protein for core neutralizing epitope of S1 protein standard [33]. 

### 2.2. Animals and Experimental Design

**Figure 1:**
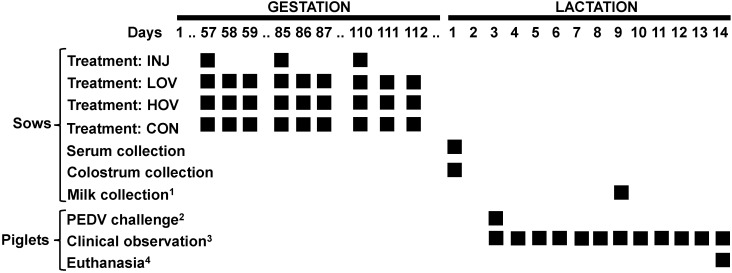
Timeline of study showing gestation and lactation periods. Injected and oral vaccines were administered to sows during gestation: INJ (injection of PEDV vaccine), LOV (low-dose oral PEDV vaccine), HOV (high-dose oral PEDV vaccine), CON (non-vaccinated controls). ^1^ Milk was collected on day 6 post-challenge. ^2^ Piglets were challenged with PEDV virus between days 3–5 of lactation. ^3^ Piglets were observed for signs of diarrhea, dehydration, and overall health for 11 d post-challenge. ^4^ Animals were euthanized on day 11 post-challenge.

Experiments were in accordance with the Institutional Animal Care and Use Committee (#VRI-S-21-1381). Sixteen Large White × Yorkshire sows were placed in a HEPA-filtered isolation room at a BSL-2 facility at Iowa State University, with a 12:12 h light/dark cycle and controlled temperature. Sows were determined to be free from PEDV by analysis of fecal material using Real PCR PEDV/PDCoV Multiplex RNA Mix (Idexx Laboratories, Westbrook, Maine), and by a serum neutralizing assay (PEDV cytometry-based high-throughput neutralization test assay) as described in [34,35]. Each sow was given two ear tags to be uniquely identified and then artificially inseminated with semen collected from PIC^®^337 boars. Sows were fed a gestation/lactation diet mixed locally according to industry standards. Water was provided ad libitum to the animals. Sows were limit-fed 4 lbs/d during pregnancy, and the ration was increased by 1 lbs/d/piglet during lactation. On day 49 of pregnancy, sows were randomly allocated to one of four treatment groups (Figure 1): (1) injected vaccine (INJ; *n* = 4), (2) non-vaccinated controls (CON; *n* = 4), (3) low-dose oral vaccine (LOV; *n* = 4), and (4) high-dose oral vaccine (HOV; *n* = 4). Sows in the INJ group were injected intramuscularly with 2 mL of a commercial PEDV vaccine (Zoetis) on days 57, 85, and 110 of gestation. The vaccine contained an undisclosed concentration of killed virus, polysorbate 80, merthiolate, and gentamicin, and 4–6% aluminum hydroxide, 1% mineral oil, and <5% of sorbitan oleate. Control sows did not receive an injected or an oral vaccine. Sows in LOV and HOV groups received 1 and 1.5 kg of corn/d containing 10 mg and 50 mg of S1 antigen, respectively, during 3 × 3-day periods starting on days 57, 85, and 110 of gestation. On each vaccination day, sows were fasted for 4 h before feeding, received the S1-transformed corn at 08:00 a.m., and then returned to their normal diet 1 h later. On day 110 of gestation, sows were moved into individual farrowing crates. Average litter size was 10.75 ± 2.38 in INJ, 10.25 ± 1.92 in CON, 12.75 ± 0.83 in LOV, and 10.5 ± 0.5 in HOV sows. Colostrum was collected manually from several teats per sow within 4–6 h after the first piglet was born. In addition, serum and milk were collected from all sows on day 1 of lactation and day 6 post-challenge, respectively. 

Between days 3 and 5 of lactation, each piglet received via intragastric route 10 mL suspension containing active PED virus obtained from Dr. Jianqiang Zhang at Iowa State University (ISU batch #PEDV USA/NC/49469/2013 at the titer of 10^4^ TCID50/mL). Following the viral challenge, piglets were monitored for 11 d for diarrhea, dehydration, and overall health, and each clinical parameter was scored according to the following rubric: Diarrhea: 0 = normal, 1 = loose and pasty, 2 = watery; Dehydration: 0 = no dehydration, 1 = mild, spine prominent, 2 = sever, spine prominent, rib cage and waist evident when viewed from above, abdomen tucked up when viewed from the side; General health: 0 = normal, 1 = lethargic, 2 = vomiting, 3 = comatose. A disease severity index was calculated daily for each litter by dividing the sum of all clinical scores by the total litter size. Mortality rate for each litter was calculated on day 11 post-challenge according to the formula: total number of piglets alive/total number of piglets challenged. Piglets were euthanized on day 11 post-challenge (d 14–16 of lactation) by injecting 10 mL of pentobarbital sodium (4 mg/kg; Zoetis) in the jugular vein, followed by exsanguination of the animals. 

### 2.3. Neutralizing Antibodies and Cytokines in Serum and Milk

Colostrum and serum samples collected immediately after farrowing, and milk samples collected on day 6 post-challenge were sent to South Dakota State University Diagnostics Laboratory (Brookings, South Dakota) for quantification of neutralizing antibodies (NABs). Briefly, Vero-76 cells were seeded onto 96 well microplates and cultured for 3–4 d. Serum and milk samples were added to the cells in serial 1:2 dilutions and PEDV virus stock was added at approximately 100 focus-forming units/well. After overnight incubation in Gibco Minimum Essential Media with 0.1% trypsin, cells were fixed by the addition of 80% acetone. Next, PEDV-specific monoclonal antibody SD6-29 (Medgene Labs, Brookings, SD) conjugated with fluorescein isothiocyanate was added to the wells, and binding was assessed with fluorescent microscope. A sample was considered positive if 90% inhibition of fluorescent foci was observed and the titer was reported as the highest dilution that has ≥90% inhibition. 

Sows’ serum and milk were also analyzed for 13 cytokines using a commercially available ELISA (Eve Technologies; Calgary, Canada): granulocyte-macrophage colony-stimulating factor (GM-CSF), interferon gamma, interleukin-1 alpha, interleukin-1 beta, interleukin-1 receptor antagonist, interleukin-2, interleukin-4, interleukin-6, interleukin-8, interleukin-10, interleukin-12, interleukin-18, and tumor necrosis factor alpha. Samples were run in duplicates and the average was used to calculate the level of individual cytokines based on a standard curve.

### 2.4. Statistical Analyses

Titers of neutralizing activity and cytokine levels in blood and serum were analyzed by a one-way ANOVA using a mixed model in R (v.4.1) that included the parameter measured as the response, treatment as the fixed effect, and sow body weight as the covariable. Normality of the residuals and presence of outliers was assessed, and nonnormally distributed parameters were log- or square root-transformed. Multiple comparisons were corrected using Tukey’s HSD procedure. Data are presented as means ± SD. Significant effects were considered at *p* *≤* 0.05. Piglet survivability was analyzed by logistic regression using a mixed model analysis that included frequency of alive/dead pigs as the response, treatment and age as the fixed effects, sow as the random effect, and body weight of piglets at challenge as the covariable. The analysis was performed in R using the *glmer* command of the *lme4* package [36]. Other packages such as *multcomp* [37], *emmeans* [38], and *performance* [39] were used to further analyze the model estimates. Tukey’s HSD procedure at 90% overall confidence was used to separate the treatments.

## 3. Results

### 3.1. Vaccination of Sows with the S Antigen Protects Nursing Piglets against Challenge with PED Virus

**Figure 2:**
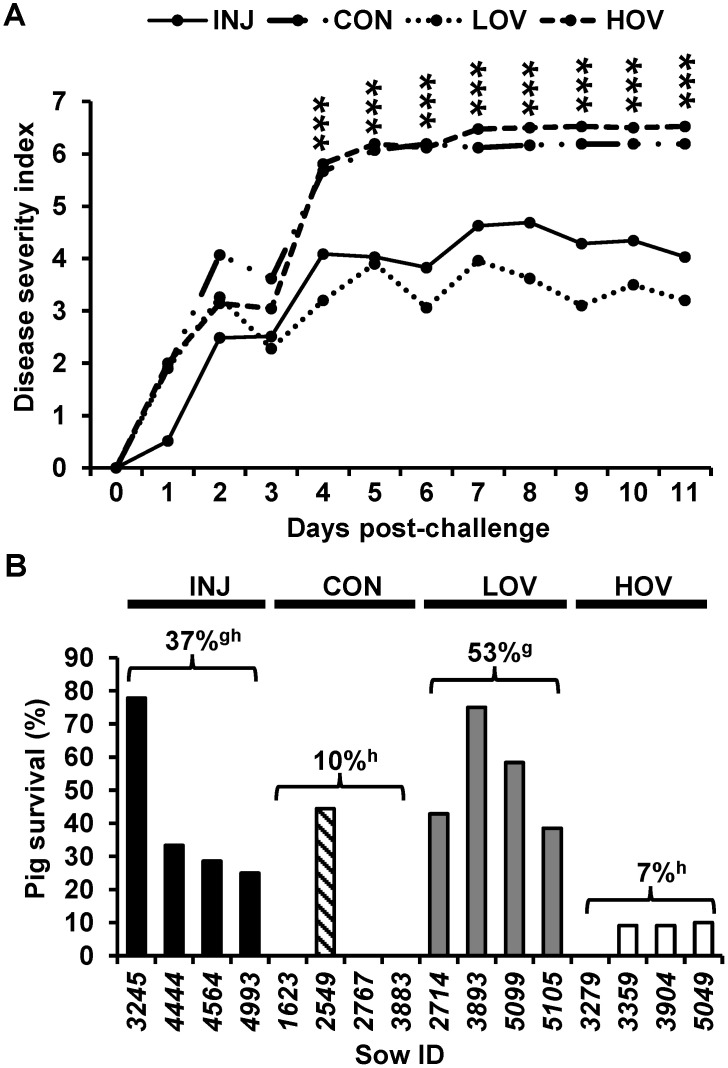
Disease severity index (**A**) and survival rate (**B**) in 14–16-day-old nursing piglets at 11 d post-challenge with active PED virus. Dams were given an injected PEDV vaccine (INJ; *n* = 4), a low-dose oral PEDV vaccine (LOV; *n* = 4), a high-dose oral PEDV vaccine (HOV; *n* = 4), or served as non-vaccinated controls (CON; *n* = 4) during pregnancy. Values are means ± SD. *** *p* *≤* 0.001; Values with different letters (g, h) *p* *≤* 0.001.

Analysis of disease index showed a significant increase in severity between days 4 and 11 post-challenge for piglets in HOV and CON sows compared with the INJ and LOV groups (*p* *≤* 0.001; Figure 2A). Mean piglet survival rates on day 11 post-challenge were 37% in INJ, 10% in CON, 53% in LOV, and 7% in HOV groups (Figure 2B). Piglet body weight at challenge (*p ≤* 0.001) and treatment (*p ≤* 0.001) were both significant predictors of survival in the generalized mixed model. Compared with HOV and CON groups, piglets from sows fed LOV had a higher survival rate (*p* *≤* 0.001; Figure 2B). Similarly, the estimated survival probability on day 11 was higher for LOV compared with HOV and CON (*p* *≤* 0.05; Table 1). 

### 3.2. Vaccination of Sows with S1 Antigen Elicits Neutralizing Antibodies Response in Serum and Milk 

**Figure 3:**
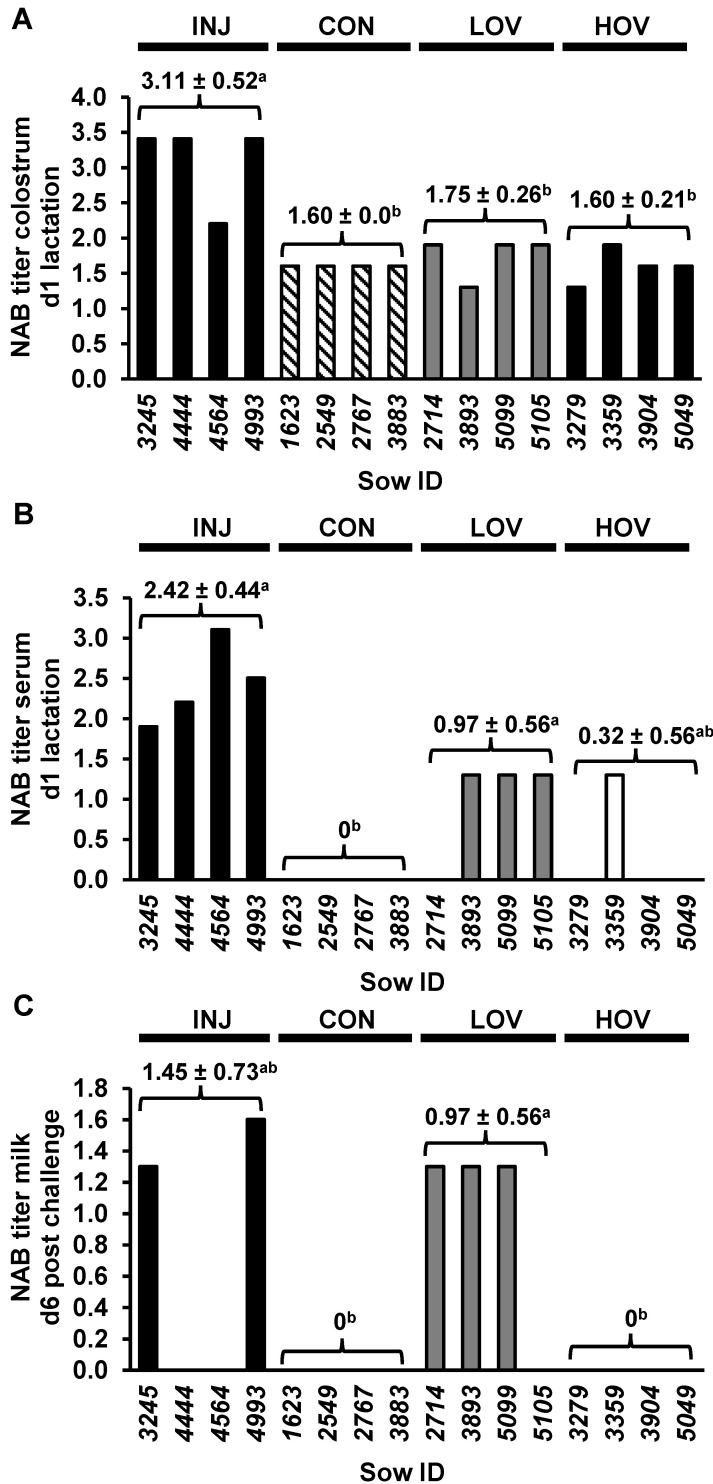
Level of NABs in colostrum (**A**), serum (**B**), and milk (**C**) of sows administered an injected PEDV vaccine (INJ; *n* = 4), a low-dose oral PEDV vaccine (LOV; *n* = 4), a high-dose oral PEDV vaccine (HOV; *n* = 4) or serve as non-vaccinated controls (CON; *n* = 4) during pregnancy. Serum and colostrum samples were collected immediately after farrowing, whereas milk samples were collected 6 d post-challenge. Results are presented as the log of the highest titer that provided a positive result, with a dilution of 20-fold being the limit of detection for a positive sample. Values are means ± SD. Values with different letters (a, b) *p* *≤* 0.05.

Immunologically, the spike (S) protein of coronavirus is the main target of virus-specific NABs, and vaccination to elicit NABs against PEDV has been considered to be the key for the prevention and control of PED [7,40,41,42]. Therefore, we measured NAB titers in sows’ serum and colostrum at farrowing, and in milk samples on day 6 post-challenge. NAB titters in colostrum were higher in INJ compared with LOV, HOV, and CON sows (*p <* 0.05; Figure 3A). NAB titers in serum were higher in INJ and LOV compared with CON (*p <* 0.05; Figure 3B), whereas NAB titers in milk increased in LOV compared with CON and HOV (*p <* 0.05; Figure 3B). 

### 3.3. Administration of S1 Antigen Effects the Level of Cytokines in Sows’ Sera and Milk 

To investigate the effect of S protein on cytokine levels in the sows, we analyzed levels of 13 cytokines in both sera and milk. There was a decrease in the level of GM-CSF in sera from LOV and INJ compared to CON (*p* *≤* 0.05; Table 2). Similarly, levels of GM-CSF were lower in milk from LOV and INJ sows compared to CON (*p* *≤* 0.05; Table 2). None of the other cytokines were statically significant between treatments.

### 3.4. NAB and GM-CSF in Milk Are Correlated with Protection in Piglets Challenged with PEDV 

Because of the widespread health and economic problems associated with COVID-19, there has been unprecedented effort to find a correlate of immune protection for SARS-CoV-2. The ability to correlate specific biomarkers with protection to the pathogen has proven to be a useful approach to identify the efficacy of vaccines without the need for a challenge, as well as to aid in the understanding the mechanism of disease. Therefore, we correlated NAB titers and cytokines in sow serum and milk against piglet survivability across all treatment groups. As it is known that older and larger pigs show fewer symptoms, we also looked for a correlation with the body weight of the piglets at challenge. Data are presented in Table 3.

Using a 5% significance level, we found piglet body weight, milk GM-CSF, and milk NABs significantly correlated with pig survival (*p ≤* 0.05; Table 3). Adjusting for the other predictors, each increase of one standard deviation in challenge weight was associated with multiplying the odds of survival by 1.96–6.11. Each increase of one standard deviation in milk GM-CSF was associated with multiplying the odds of survival by 0.05–0.72. The presence of milk NABs was associated with multiplying the odds of survival by 3.71–65.89 compared to the absence of milk NABs.

There may be different mechanisms of protection involved depending on the dose and the type of administration that could affect the elicitation of NABs or cytokine levels that in turn could have a combined effect on survival. Interestingly, LOV sows had GM-CSF < 436 pg/mL, whereas CON sows without surviving piglets showed GM-CSF > 1193 pg/mL (Table 4). The only control sow with piglets surviving the challenge was also the only sow to have a low GM-CSF (291 pg/mL). 

## 4. Discussion

In this study, we demonstrated that maize-produced, orally delivered S antigen administered to pregnant sows provides passive immunity to suckling piglets. Following a challenge with PED virus, we observed higher survival rates of newborn piglets from litters fed a low dose S antigen compared to control animals. Therefore, our study represents a practical approach to develop a cost-effective, orally delivered, heat-stable, and efficacious vaccine against PEDV. Our data also corroborate earlier work in which oral feeding of the S protein to 2-month-old piglets increased serum NAB titers after challenge with PEDV [33]. Similarly, administration a maize-produced vaccine candidate against transmissible gastroenteritis virus (TGEV) provided an increase in NAB in colostrum and milk compared to the injected vaccine or control animals [29]. Interestingly, the high oral dose of the vaccine candidate provided less protection in piglets compared with the low dose. We have observed a similar effect in TGEV vaccinated pigs, in which lower dose of the TGEV vaccine provided full protection against the virus while higher doses led to reduced protection [28]. Although speculative, it is possible that this effect is due to the onset of oral tolerance against the vaccines; nonetheless, further studies are needed to confirm this theory. 

The PED virus belongs to the same family of a positive strand enveloped RNA virus as MERS-CoV (Middle East Respiratory Syndrome Coronavirus), SARS-CoV-1, and SARS-CoV-2 (Severe Acute Respiratory Syndrome Coronavirus 1 and 2). In light of the ongoing SARS-CoV-2 pandemic, the elucidation of cellular mechanisms of protection against coronaviruses remains critical. Previous studies of vaccines against MERS-CoV and SARS-CoV-1 have shown that both humoral and cellular immune responses are important to elicit protection [43]. In this regard, both serum and milk NABs were increased in sows fed a low dose of an oral vaccine compared with controls, whereas NAB titers in colostrum did not differ between HOV, LOV, and CON sows, and were significantly higher in animals receiving the injected vaccine. The presence of NAB titters in CON colostrum is likely due to some background NAB activity in our assay, due to extremely elevated concentration of immunoglobulins in colostrum. In fact, NABs were not present in serum of CON sows. In addition, the increase in colostrum NABs in INJ vs. both HOV and LOV sows is likely due to the inherent immunoglobulin composition of pig colostrum. IgGs, which are induced by injected vaccines, represent the main immunoglobulins in sow serum and colostrum, whereas IgAs, potentially induced by our oral vaccine in gut mucosa, constitute the major immunoglobulins in sow milk but have very low levels in colostrum [44,45]. Of note, NAB colostrum titers in sows decline rapidly after farrowing [46], and therefore, it is unlikely that they had a significant effect on protection against PEDV in our study. 

Results from this and previous studies [47] indicate that serum NAB titers do not correlate with PEDV protection. Conversely, we and others have shown NABs in milk to be inversely correlated with piglet mortality rate [40]. It is important to remark that this effect is not a causal relationship, and there is likely more than one mechanism involved in providing protection in nursing piglets. For example, one out of four nonvaccinated sows showed a 45% piglet survival rate compared to no surviving pigs in the other three sows, highlighting both natural variability beteen animals and the presence of additional factors involved in PEDV protection. In this regard, previous studies have documented an active cytokine transfer from colostrum/milk to neonates [48], and maternal cytokines have been linked to the physiological maturation of gastrointestinal immune system in suckling pigs [48,49]. Furthermore, recent reports have revealed that both GM-CSF and GM-CSF receptors are increased and correlated with disease severity in many inflammatory and autoimmune diseases, such as inflammatory bowel disease, multiple sclerosis, rheumatoid arthritis, and SARS-CoV-2 [50,51]. In agreement with these studies, we observed a significant decrease in serum GM-CSF levels in LOV and INJ sows compared with CON, suggesting that the S antigen may have a downregulatory effect on GM-CSF production. Interestingly, levels of GM-CSF in sow milk were inversely correlated with survival rate of suckling piglets, supporting the passive transfer of lactogenic immunity and potential immunomodulatory of GM-CSF in piglets. 

## 5. Conclusions

In summary, we demonstrated here that a mucosal-delivered vaccine candidate provides passive immunity and protection to suckling piglets. These findings present evidence that our maize-based vaccine platform can be used as a cost-effective, convenient, and an effective form of vaccination against PEDV in sows, eliminating many of the expenses and inconvenience of parentally delivered vaccines. In addition, we show correlates of protection with higher levels of NABs and lower levels of GM-CSF in sows’ milk, corresponding to a higher survivability of the nursing piglets. Further studies looking at the cellular and histological level of piglets’ mucosa are warranted.

## Figures and Tables

**Table 1:** Statistical probability of survival of 14–16-day-old nursing piglets at 11 d post-challenge with active PED virus.

Treatment	Probability	Groupings
INJ	0.26	ab
CON	0.09	a
LOV	0.56	b
HOV	0.08	a

Dams were given an injected PEDV vaccine (INJ; *n* = 4), a low-dose oral PEDV vaccine (LOV; *n* = 4), a high-dose oral PEDV vaccine (HOV; *n* = 4), or served as non-vaccinated controls (CON; *n* = 4) during pregnancy. The probabilities were computed assuming piglets of average weight. Values with different letters (a, b) *p ≤* 0.05.

**Table 2:** Cytokine levels in serum and blood of pregnant sows administered an injected PEDV vaccine (INJ; *n* = 4), a low-dose oral PEDV vaccine (LOV; *n* = 4), a high-dose oral PEDV vaccine (HOV; *n* = 4), or serving as non-vaccinated controls (CON; *n* = 4).

Cytokine	INJ	CON	LOV	HOV
GM-CSF	200.1 ^a^ ± 183.7	1128.1 ^b^ ± 547.6	159.3 ^a^ ± 179.1	381.2 ^ab^ ± 238.4
IFNγ	3903.7 ± 4063.2	10694.7 ± 1352.6	4567.7 ± 4689.0	5448.4 ± 5079.1
IL-1α	158.8 ± 104.6	472.1 ± 145.6	325.7 ± 186.9	262.2 ± 190.4
IL-1β	1624.8 ± 1095.4	4874.9 ± 2181.3	3092.4 ± 1972.9	3583.5 ± 3039.3
IL-1RA	938.9 ± 619.6	2992.1 ± 874.8	2290.8 ± 1451.3	2330.7 ± 1992.9
IL-2	1371.6 ± 979.2	4386.1 ± 1417.7	2959.8 ± 1959.1	3026.7 ± 2549.3
IL-4	4404.2 ± 3329.9	15752.0 ± 5540.5	10763.5 ± 7541.7	10326.9 ± 8721.3
IL-6	468.3 ± 368.8	1138.9 ± 501.4	700.3 ± 554.9	1274.6 ± 1185.8
IL-8	8710.6 ± 5239.0	6812.2 ± 3008.8	8509.7 ± 3806.0	8254.3 ± 1823.0
IL-10	2135.5 ± 1565.5	7833.4 ± 3245.6	5569.7 ± 3664.9	4791.4 ± 4135.0
IL-12	589.2 ± 439.1	1702.4 ± 535.8	1196.6 ± 763.0	1157.8 ± 968.0
IL-18	2855.0 ± 2099.3	7994.6 ± 3349.0	5366.2 ± 3520.9	6667.1 ± 5481.6
TNFα	421.7 ± 501.5	518.1 ± 273.1	225.0 ± 271.3	703.5 ± 623.6

Blood and milk samples were collected on day 1 of lactation and 6 d post-challenge, respectively. Results are presented in pg/mL. Values are means ± SD. Values with different letters (a, b) *p* *≤* 0.05. GM-CSF: granulocyte-macrophage colony-stimulating factor; IFNγ: interferon gamma; IL-1α: interleukin-1 alpha; IL-1β: interleukin-1 beta; IL-1RA: interleukin-1 receptor antagonist; IL-2: interleukin-2; IL-4: interleukin-4; IL-6: interleukin-6; IL-8: interleukin-8; IL-10: interleukin-10; IL-12: interleukin-12; IL-18: interleukin-18; TNFα: tumor necrosis factor alpha.

**Table 3:** Correlation between piglet survival, piglet body weight at challenge, and levels of granulocyte-macrophage colony-stimulating factor (GM-CSF) and neutralizing antibody (NABs) titers in sow milk and serum.

Predictor	*p*-Value	Odds Ratio 95% Lower Limit	Odds Ratio 95% Upper Limit
Standardized Weight	*≤*0.001	1.96	6.11
Standardized Milk GM-CSF	0.02	0.05	0.72
Standardized Sera GM-CSF	0.27	0.68	4.01
Milk NABs	*≤*0.001	3.71	65.89
Sera NABs	0.38	0.11	2.18

Variables with *p* ≤ 0.05 are significant predictors of piglet survivability. Cytokine levels were standardized such that a value of one indicates a GM-CSF level one standard deviation above average. Milk NAB titers greater than or equal to 40 and sera NAB titers were greater than or equal to a titer of 20 were used as the cut-off values.

**Table 4:** Levels of granulocyte-macrophage colony-stimulating factor (GM-CSF) in sow milk on day 6 post-challenge, and percentage of piglets alive on day 11 post-challenge in sows administered an injected PEDV vaccine (INJ; *n* = 4), a low-dose oral PEDV vaccine (LOV; *n* = 4), a high-dose oral PEDV vaccine (HOV; *n* = 4), or serving as non-vaccinated controls (CON; *n* = 4) during gestation. To distinguish from the figures, keep the sow IDs Italic.

Sow ID	Treatment	Milk GM-CSF (pg/mL)	Piglets Surviving (%)
*3245*	INJ	219	78
*4444*	INJ	92	17
*4564*	INJ	489	28
*4993*	INJ	0	25
*1623*	CON	1193	0
*2767*	CON	1201	0
*2549*	CON	291	44
*3883*	CON	1828	0
*2714*	LOV	436	43
*3893*	LOV	199	75
*5099*	LOV	0	38
*5105*	LOV	0	58
*3279*	HOV	557	0
*3359*	HOV	608	9
*3904*	HOV	0	9
*5049*	HOV	358	10

## Data Availability

Data is contained within the article.

## Abbreviations

PEDV: Porcine epidemic diarrhea virus; COE: core neutralizing epitope; GM-CSF: granulocyte macrophage-colony stimulating factor; NABs: neutralizing antibodies; TNF-α: tumor necrosis factor alpha; S: spike protein of coronavirus.
